# Influence of parental factors on adolescents’ transition to first sexual intercourse in Nairobi, Kenya: a longitudinal study

**DOI:** 10.1186/s12978-015-0069-9

**Published:** 2015-08-21

**Authors:** Chinelo C. Okigbo, Caroline W. Kabiru, Joyce N. Mumah, Sanyu A. Mojola, Donatien Beguy

**Affiliations:** Department of Maternal and Child Health, Gillings School of Global Public Health, University of North Carolina, Chapel Hill, NC USA; African Population and Health Research Center, APHRC Campus, Manga Close, Off Kirawa Road, Nairobi, Kenya; Department of Sociology and Institute of Behavioral Science, University of Colorado Boulder, Boulder, CO USA

**Keywords:** Parental monitoring, Parent-child communication, Parental discipline, Adolescents, Sexual debut, Urban slums

## Abstract

**Background:**

Several studies have demonstrated a link between young people’s sexual behavior and levels of parental monitoring, parent-child communication, and parental discipline in Western countries. However, little is known about this association in African settings, especially among young people living in high poverty settings such as urban slums. The objective of the study was to assess the influence of parental factors (monitoring, communication, and discipline) on the transition to first sexual intercourse among unmarried adolescents living in urban slums in Kenya.

**Methods:**

Longitudinal data collected from young people living in two slums in Nairobi, Kenya were used. The sample was restricted to unmarried adolescents aged 12–19 years at Wave 1 (weighted *n* = 1927). Parental factors at Wave 1 were used to predict adolescents’ transition to first sexual intercourse by Wave 2. Relevant covariates including the adolescents’ age, sex, residence, school enrollment, religiosity, delinquency, and peer models for risk behavior were controlled for. Multivariate logistic regression models were used to assess the associations of interest. All analyses were conducted using Stata version 13.

**Results:**

Approximately 6 % of our sample transitioned to first sexual intercourse within the one-year study period; there was no sex difference in the transition rate. In the multivariate analyses, male adolescents who reported communication with their mothers were less likely to transition to first sexual intercourse compared to those who did not (*p* < 0.05). This association persisted even after controlling for relevant covariates (OR: ≤0.33; *p* < 0.05). However, parental monitoring, discipline, and communication with their fathers did not predict transition to first sexual intercourse for male adolescents. For female adolescents, parental monitoring, discipline, and communication with fathers predicted transition to first sexual intercourse; however, only communication with fathers remained statistically significant after controlling for relevant covariates (OR: 0.30; 95 % C.I.: 0.13–0.68).

**Conclusion:**

This study provides evidence that cross-gender communication with parents is associated with a delay in the onset of sexual intercourse among slum-dwelling adolescents. Targeted adolescent sexual and reproductive health programmatic interventions that include parents may have significant impacts on delaying sexual debut, and possibly reducing sexual risk behaviors, among young people in high-risk settings such as slums.

## Background

Adolescence, which is marked by physical, psychosocial, and intellectual development, prepares young people for adult roles and responsibilities [1]. This period is characterized by experimentation as young people define their values and seek independence. Unfortunately, such experimentation also drives adolescents to engage in risky behaviors. Indeed, many behaviors that lead to illness or premature death later in life (e.g., sexual risk behaviors, substance use, unhealthy diet, and physical inactivity) are often initiated during adolescence [1]. Sexual risk behavior is defined as any behavior that increases a person’s risk of unintended pregnancy and/or sexually transmitted infections (STIs) including human immunodeficiency virus (HIV) infection [2, 3]. These sexual risk behaviors include early age at sexual debut, multiple sexual partners, unprotected sexual intercourse, and sexual intercourse while under the influence of alcohol or drugs [3–5]. Sexual risk behaviors in adolescence are a cause of concern not only because they predispose adolescents to adverse health outcomes but also because they have been shown to be predictors of sexual risk behaviors later in life [1]. According to the World Health Organization (WHO), adolescent pregnancy increases the risk of maternal and child mortality [6]. The second most common cause of mortality among female adolescents (ages 15–19) is pregnancy-related complications, often from unsafe abortion [6]. In addition, babies born to adolescent mothers are at a higher risk of dying compared to those born to older mothers [6]. Furthermore, adolescents have a huge burden of STIs including HIV; approximately 1 in 20 sexually active adolescents contract an STI every year [7]. Despite a global decline in mortality due to HIV, HIV mortality among adolescents is on the increase; it is estimated that 2 million adolescents are living with HIV in 2012, and a disproportionate number of them live in sub-Saharan Africa [1]. African youth living in urban slums may be especially vulnerable to HIV infection in the context of extreme poverty, limited economic means, and sexual violence [8].

The importance of parental involvement is often underscored in risk-reduction efforts targeted towards adolescents because parents/guardians are in regular contact with their children and, therefore, presumably in a good position to shape and influence young people’s behaviors [4, 9]. Consequently, the influence of parental factors on adolescent sexual behavior has been the subject of numerous studies. These studies have demonstrated a link between young people’s sexual behavior and levels of parental monitoring, parent-child communication, and parental discipline [10–14]. However, the majority of the studies have been conducted in Western countries. Little is known about this association in sub-Saharan African settings, and informal/slum settlements in particular. Previous studies have shown that young people living in high poverty settings, such as urban slums, engage in sexual intercourse much earlier and have more sexual partners than their peers that live in wealthier households [15–17]. However, with the exception of a few studies [16, 18], the role of parental factors in high-risk urban settings in sub-Saharan Africa has been understudied. This study fills the gap in the current literature by examining the role of parenting on transition to first sexual intercourse among adolescents living in two urban slums in Nairobi, Kenya’s capital city. The implications of study findings for policies and programs are also highlighted.

This study focused on three parenting factors that have been found to have an impact on adolescents’ sexual risk behavior: parental monitoring, parent-child communication, and parental discipline. Parental monitoring is the deliberate effort by parents to control whom their children are with and where they spend their free time; however, from the children’s perspective, it is often defined as adolescents’ perception of their parents’ knowledge about whom they are with and where they are spending their free time [9, 10]. A high correlation between the parents’ and children’s report of parental monitoring has been documented [19]. High levels of parental monitoring are shown to be associated with a delay in sexual debut and low involvement in sexual risk behaviors [4, 9, 10, 12–14, 20]. Age and sex of the adolescent have also been found to modify parental monitoring so defined. In general, parents tend to adjust their monitoring practices as the adolescents get older to allow for more independent decision-making. Consequently, older adolescents report lower levels of parental monitoring compared to younger adolescents [12, 21, 22]. In addition, female adolescents have been shown to perceive more parental monitoring and discipline compared to their males counterparts [12, 21–23]. This can be attributed to the existence of a “double standard” in the sexual expectations of the adolescents based on their sex – sexual activity is tolerated for males but frowned at for females as found in many African communities [24]. School enrollment also affects the level of parental monitoring as studies have shown that parents tend to monitor students who are enrolled in school more than those who are not [25, 26]. Higher parental monitoring of in-school adolescents may be attributed to the high opportunity cost of having a child drop out of school or perform poorly in their academics. Additionally, previous research indicates that the combined effect of religion and supportive parenting practices increases the adolescents’ perception of social support and deters their engagement in risky behaviors [27]. Likewise, religious adolescents in urban poor settings have been shown to be less likely to engage in delinquent behaviors including sexual risk behaviors [27–29].

Positive parent-child communication is known to be protective against risk behaviors in adolescence [10, 12, 20]. However, results of studies investigating the link between parent-child communication about sex-related issues and young people’s sexual risk behaviors have been mixed. For example, a study conducted in the United States found that positive general communication between Black and Hispanic mothers and their adolescents was associated with less frequent sexual intercourse and fewer sexual partners [20]. Similarly, a 2009 study in four African countries found that parent-child communication about sexual activity was associated with delayed sexual debut in female adolescents [9]. However, several studies have found the opposite association to be true: parent-child communication about sex-related issues was found to be associated with earlier sexual initiation among adolescents especially for males [26, 30]. This discrepancy could be due to cultural differences as the studies where parent-child communication was reported to be protective were among minority groups and African communities where communication about sexuality is sparse between parents and children. In some traditional African communities, direct parent-child communication about sexuality is often considered a “taboo” and in other communities, young people receive such information during traditional rites of passage and, often, through someone other than their parents [31, 32]. However, with the decline in traditional cultural practices, rapid urbanization, and the breakdown of extended family ties in many urban settings in sub-Saharan Africa, parents are forced to assume the responsibility of imparting sexuality education. There is evidence that in African settings, parent-child discussions on matters relating to sex occur late (usually after sexual debut); often because parents feel that introducing discussions of sexuality too early will increase curiosity about sex and possibly lead to experimentation [31]. Consequently, communication between parents and their children about sexual matters may commence only after a young person is perceived to have engaged in sexual intercourse.

Adolescents who perceive that their parents disapprove of sexual activity and anticipate parental discipline are less likely to engage in sexual activity leading to a delay of sexual debut [20, 33]. A study conducted among Lao/Mien adolescents found that parental discipline, which included punishment, taking activities away, sending the adolescents to their room, yelling or scolding, slapping or spanking, and/or making the adolescents feel shameful when they behaved badly, prevented adolescents from engaging in risky sexual activity for both boys and girls [26]. However, parental discipline seems to have a curvilinear relationship with adolescents’ engagement in sexual risk behaviors – adolescents who perceived the least parental discipline have the highest sexual risk behaviors followed by those who perceive the strictest parental discipline while those who perceive moderate parental discipline have the least sexual risk behaviors [34]. It is posited that setting behavioral limits without clarifying expectations by parents may have an adverse effect not just on their self-esteem, but also on adolescent sexual risk behavior [28].

This study, therefore, sought to answer two research questions: 1) is adolescents’ perception of parental monitoring, parent-child communication, and parental discipline associated with their transition to first sexual intercourse within the one-year study period?; and 2) do the characteristics of the adolescent modify the relationship between parental factors and adolescents’ transition to first sexual intercourse? Based on the findings from the literature, we hypothesized that high levels of parental monitoring, greater parent-child communication about general matters, and high levels of parental discipline would be associated with a delay in sexual debut among urban slum-dwelling adolescents. We also postulated that the characteristics of the adolescents such as age, sex, school enrollment, peer models for risk behavior, delinquency, and religiosity would modify the relationship between the parental factors and the adolescents’ transition to first sexual intercourse.

## Methods

### Study design and procedures

We drew on data collected under the Transitions to Adulthood (TTA) project, which sought to identify protective and risk factors in the lives of adolescents growing up in two informal settlements in Nairobi (Korogocho and Viwandani) and to examine how those factors influence their transition to adulthood. Viwandani is located in the industrial area of Nairobi and thus, attracts a youthful, highly mobile population that is seeking employment in nearby industries. Korogocho is a more stable settlement with a median duration of stay of 16 years for the current population compared to seven years in Viwandani. Both slums however, are characterized by high levels of unemployment, crime, substance abuse, limited and often, inadequate schooling facilities, and lack of recreational facilities. The Nairobi Urban Health Demographic Surveillance System (NUHDSS) covers a Demographic Surveillance Area (DSA) that spans the Korogocho and Viwandani slums in Nairobi City, Kenya. The NUHDSS provides a platform for studying the associations between urbanization, poverty, and health, and serves as a tool for monitoring and evaluating the impact of health intervention programs on health outcomes of the study population. Currently, the NUHDSS covers approximately 60,000 people living in 22,000 households in Korogocho and Viwandani. The surveillance involves visits to all the households every four months to update information on demographic and health indicators.

The TTA project was based on a random sample of adolescents within the households in the study areas using records of residents in the NUHDSS in 2007. Between October 2007 and June 2008, a random sample of about 4,058 young people aged 12–22 years was interviewed. Approximately one year later (March-August 2009), an updated questionnaire was administered to the same cohort. About 2,674 adolescents were re-interviewed at the follow-up survey; the high attrition rate was due to out-migration and the 2008–2009 post-election violence that occurred in several urban communities after the 2008 general elections in Kenya. This paper includes adolescents aged 12–19 years who were interviewed at both the baseline (Wave 1) and follow-up (Wave 2) surveys. Since the primary aim of the research presented in this article was to examine the effect of parental factors on adolescent sexual transition, the sample was restricted to adolescents who had never been married by Wave 2 [*n* = 1927]. The rationale is that unmarried adolescents are more likely to be wards of their parents and hence, have the chance to experience the parental factors.

To address selection bias, the characteristics of adolescents who were interviewed in both waves were compared to those interviewed only in Wave 1. Adolescents who were interviewed in both waves did not differ from those interviewed only in Wave 1 by sex. However, a greater proportion of adolescents interviewed in both waves were younger, lived in Viwandani versus Korogocho, lived with at least one parent, were currently enrolled in school, and had never had sexual intercourse [data not shown]. To account for these differences in subsequent analyses, a logistic regression model was run using response status at Wave 2 as the outcome variable, and key socioeconomic and demographic characteristics at Wave 1 as explanatory variables. The probability of responding at each wave was calculated for every individual in the data using logistic regression, controlling for characteristics that were found to predict non-response. The analytical weight for the observations was obtained by taking the inverse of the predicted probability. The computed weights were re-adjusted to approximately add up to the expected sample size. The overall weight for Wave 2 was obtained by taking the product of Wave 1 and Wave 2.

The study instrument included questions on living arrangements and the nature of interactions with parents, guardians, and peers; involvement in youth groups (e.g., religious and social groups); and involvement in risky behaviors (e.g., early sexual debut and delinquency). The complete questionnaire was translated from English to Swahili and administered in Swahili, the national language. Respondents were requested to give signed informed consent; for respondents aged 12–17 who were living with parents or guardians, signed informed consent was also requested from their parents or guardians. The data for this study were collected in 2007/08 (Wave 1) and 2008/09 (Wave 2). The study received ethical approval from the Kenya Medical Research Institute’s ethical review board.

### Measures

#### Outcome variable

The outcome variable was sexual intercourse status (transition to first sexual intercourse) at Wave 2. For bivariate analysis, adolescents were grouped into three categories: “Virgins” if they have never had sexual intercourse (both Waves 1 and 2); “Non-virgins” if they had sexual intercourse at Wave 1; or “Transitions” if they hadn’t had sexual intercourse at Wave 1 but had by Wave 2. For the logistic regression analyses, only the subset of adolescents reporting no sexual intercourse at Wave 1 was used [*n* = 1507]. The outcome variable was grouped into whether or not the adolescent transitioned to first sex between Waves 1 and 2 (coded ‘0’ for no or ‘1’ for yes).

#### Explanatory variables

The three parental factors at Wave 1, parental monitoring, parent-child communication, and parental discipline, were the explanatory variables. Parental monitoring was assessed using six questions that asked study participants whether they perceived that their parents knew where they were in the evenings of week days; who they were with in the evenings of week days; where they were during the weekends; who they were with during the weekends; who their friends were; and what they did with their free time. Responses were graded on a 3-point scale – 1 (never), 2 (sometimes), and 3 (always). A continuous parental monitoring index was constructed using standardized values of individual items all scored in the positive direction using the “standardize” function in Stata 13 (Cronbach’s alpha = 0.90). Given that 5 % of adolescents were not living with a parent, scores on the parental monitoring index were then grouped into three categories: “low/does not live with parent”, “medium”, or “high” parental monitoring using the “cut” function in Stata.

Communication with parents (mother figure, father figure) was assessed using a set of three questions that measured the extent to which the respondent: 1) felt that their mother or father figures taught him/her things he/she does not know; 2) shared secrets with his/her mother or father figures; and 3) that his/her mother or father figures tried to help him/her. The responses were graded on a 5-point scale, which are 1 (never), 2 (sometimes), 3 (half the time), 4 (most of the time), and 5 (all the time). Continuous parent-child communication indices were created using standardized values of individual items all scored in the positive direction (communication with mother figure: Cronbach’s alpha = 0.45; communication with father figure: Cronbach’s alpha = 0.48). Scores were grouped into three categories: “no”, low”, and “high” communication.

Parental discipline was assessed using two questions that asked the adolescents whether their parents/guardians: 1) verbally reprimand (scolding); and/or 2) spank them when they do something wrong. Responses were graded on a 5-point scale ranging from 1 (never/not living with parents/guardian) to 5 (always). Item scores were recoded into 4 categories for “not living with parents”, “never”, “occasionally” and “frequently”. For the multivariate analyses, we created binary variables with response categories ‘0’ for no or ‘1’ for yes.

#### Control variables

We controlled for several factors found to be associated with adolescent sexual risk behavior in various settings [12, 21, 22, 35]: adolescents’ age, sex, residence, school enrollment, peer models for risk behavior, delinquency, and religiosity (all measured at Wave 1). School enrollment status was determined by asking the adolescents whether they were currently enrolled in school. School enrollment was categorized as “in” versus “out of school”. Peer models for risk behavior was measured using four items: the proportion of friends who: get good grades in school, participate in sports or other school activities, attend church/mosque, and want to go to secondary school, university, or college (Cronbach’s alpha = 0.94). Possible responses are none of them (coded 0), some of them (1), most of them (2), don’t know, or not applicable. “Don’t know” and “not applicable” responses were treated as missing values. Delinquent behavior was assessed using a set of seven items that measured the extent to which youth engaged in the following behaviors in the four months preceding the survey: staying away from home for at least one night without parental permission; starting a fight with peers; taking or trying to take something belonging to someone else without their knowledge; carrying a knife, gun, or other weapon; hitting or threatening to hit a peer or adult; delivering or selling drugs; and delivering or selling alcohol. Reponses ranged from 0 = never; 1 = once; 2 = more than once. A composite index was then derived from standardized values; an internal consistency was measured using the Cronbach’s alpha (α = 0.69). Religiosity was a composite measure created using five items that assessed the frequency of participation in religious services (i.e., how many times have you gone to religious services during the past one month? Response options: never, 1 time, 2–3 times, 4 times, more than 4 times) and the importance of relying on religious teachings and beliefs, believing in God, and prayer in one’s life (e.g., how important is it to you to be able to rely on religious teachings when you have a problem? Response options: not important, somewhat important, important, and very important (Cronbach’s alpha = 0.93).

### Analyses

Quantitative data analyses were performed using Stata 13 [36]. Bivariate analyses (chi-square, cross-tabulations, ANOVA, and t-tests) were conducted to assess bivariate associations between sexual intercourse status and the independent and control variables, as well as examine age differences by sex. Chi-square tests were used to determine the statistical relationships between the categorical variables in the cross tabulations. Multivariate logistic regression models were run in which baseline (Wave 1) measures of parental and control variables were used to predict the likelihood of making the transition to first sexual intercourse between the two waves. Two sets of models were run: the first included only parental factors, while the second model added the control variables to assess the effect of these variables on the base parental model. The analyses were run separately for males and females. All analyses were based on values weighted for non-response at Wave 2.

## Results

### Descriptive analysis

Table 1 provides the socio-demographic description of the respondents stratified by sex. Of the 1,927 adolescents included in this study, 52 % were males, 43 % were aged less than 15 years, 52 % lived in Viwandani, 95 % lived with at least one parent, and 77 % were enrolled in school. Seventy-two percent of respondents reported that they had never had sexual intercourse in both waves, while 6 % reported their first sexual intercourse between the two waves.

**Table 1:** Respondents’ socio-demographic characteristics, by sex

	Total (*N* = 1927)	Males (*n* = 1002)	Females (*n* = 925)
Baseline mean age in years (SE)	15.2 (0.05)	15.3 (0.07)	15.2 (0.07)
Baseline age group (%)			
12–14 year olds	42.7	42.0	43.4
15–17 year olds	36.3	37.1	35.5
18–19 year olds	21.0	20.9	21.1
Baseline residence (%)			
Korogocho	48.4	47.0	50.1
Viwandani	51.6	53.1	50.0
Baseline living arrangements (%) *			
With parent(s)	94.8	91.9	98.2
Without parents	5.2	8.1	1.8
Baseline school enrollment (%)			
In school	77.3	78.8	75.7
Out of school	22.7	21.2	24.3
Sexual intercourse status at Wave 2 (%)			
Non-virgins	21.9	23.9	19.6
Transitions	6.0	5.9	6.0
Virgins	72.2	70.2	74.4

Notes: Estimates are weighted; SE = Standard Error

*Significant difference at *p* < 0.05 between males and females based on chi-square tests

### Bivariate analysis

Table 2 summarizes the results of the bivariate analysis by sexual intercourse status and sex of the respondents. For both males and females, the majority of the non-virgins were in the oldest age group. The mean age for non-virgins was 17.3 years for both males and females compared with 14.5 years and 14.6 years, for male and females virgins respectively. A majority of those who made the transition to first sexual intercourse were also in the older age group. Most of the virgins (99.7 % for females and 97.2 % for males) were living with at least one parent. Ninety-four percent of female non-virgins and 77 % of male non-virgins were living with at least one parent. Eighty-nine percent of both male and female adolescents classified as virgins were enrolled in school compared to fewer than 34 % of females and 49 % of males categorized as non-virgins.

**Table 2:** Descriptive statistics by sexual intercourse status and by sex

	Males			Females		
	Non-virgins (*n* = 239)	Transitions (*n* = 59)	Virgins (*n* = 704)	Non-virgins (*n* = 181)	Transitions (*n* = 56)	Virgins (*n* = 688)
Baseline sociodemographic characteristics						
Mean age (SD) in years^a, b^	17.3 (1.74)	16.3 (2.03)	14.5 (2.04)	17.3 (1.69)	16.4 (1.76)	14.6 (2.07)
Residence ^b^ (%)						
Korogocho	54.1	44.7	44.7	62.5	52.7	46.6
Viwandani	45.9	55.3	55.3	37.5	47.3	53.4
Living arrangement^a, b^ (%)						
With parent(s)	76.7	89.6	97.2	93.6	94.1	99.7
School enrollment^a, b^ (%)						
In school	48.5	62.5	90.4	33.5	51.9	88.7
Baseline psychosocial controls						
Mean (SD) peer models for risk behavior^a, b, c^	0.51 (1.21)	0.19 (1.06)	−0.06 (0.93)	0.35 (1.16)	−0.01 (0.86)	−0.19 (0.79)
Mean (SD) delinquency^a, b, c^	0.41 (1.04)	0.02 (0.59)	0.01 (0.54)	0.16 (0.67)	−0.11 (0.40)	−0.17 (0.36)
Mean (SD) religiosity^a, b, c^	−0.50 (1.25)	−0.26 (1.15)	0.00 (0.88)	−0.11 (0.91)	0.07 (0.69)	0.19 (0.63)
Baseline parenting variables						
Parental monitoring^a, b^ (%)						
Low/does not live with parent(s)	61.3	47.9	36.4	54.6	34.6	17.6
Medium	25.8	23.0	30.9	31.4	36.5	36.2
High	12.9	29.1	32.7	14.0	29.0	46.2
Parental discipline (scolding)^a, b^ (%)						
Not living with parents	19.6	8.9	2.3	5.3	5.9	0.1
Never	10.3	17.8	10.7	15.9	17.6	10.0
Occasionally	58.7	59.4	76.8	67.8	62.2	80.9
Frequently	11.4	13.9	10.2	11.1	14.4	9.1
Parental discipline (spanking/slapping)^a, b^ (%)						
Not living with parents	19.6	8.9	2.3	5.3	5.9	0.1
Never	62.1	68.5	44.3	72.6	74.4	50.2
Occasionally	17.4	22.6	48.9	20.9	16.1	47.4
Frequently	0.9	0.0	4.5	1.2	3.7	2.3
Communication with father figure^a, b^ (%)						
Low	42.9	32.7	34.8	43.7	30.7	37.3
High	27.9	48.4	42.7	20.0	34.7	42.0
No father figure	29.2	18.9	22.5	36.3	34.6	20.7
Communication with mother figure^a, b^ (%)						
Low	61.8	48.4	57.7	37.8	38.3	28.0
High	29.5	34.2	39.2	57.7	56.1	70.5
No mother figure	8.7	17.4	3.1	4.6	5.6	1.4

Notes: Estimates are weighted; SD = Standard Error

^a^Significant difference at *p* < 0.05 across groups for males based on chi-square tests for categorical variables and ANOVA for continuous variables

^b^Significant difference at *p* < 0.05 across groups for females based on chi-square tests for categorical variables and ANOVA for continuous variables

^c^Increasing values indicate higher levels of negative peer influence, delinquency, or religiosity

All psychosocial variables were significantly associated with sexual intercourse status at the bivariate level for both females and males. Assessing the peer models for risk behavior and delinquency, we found that males and females who were non-virgins had higher levels of negative influence and delinquency compared with those who were virgins and those who transitioned during the one-year follow-up period. A similar association was observed with religiosity. Adolescent female and male non-virgins, on average, had lower scores on the religiosity index compared with the other two groups.

Forty-six percent (46 %) of female virgins reported high parental monitoring, while only 29 % of females who transitioned to first sexual intercourse and 14 % of female non-virgins reported high parental monitoring. A similar trend was observed for males. A greater proportion of females than males reported high parental monitoring (*p* < 0.05). Although a substantial proportion of both male and female adolescents reported being occasionally scolded or spanked, adolescent males and females who transitioned from virgins to non-virgins were more likely to have reported that they had never been scolded or spanked. It should be noted that generally, spanking and/or slapping were not common forms of discipline by parents of adolescents in the slums. Among adolescents who were sexually experienced at Wave 2 (i.e. non-virgins and transitions), 47 % had initiated sexual activity by age 15 [data not shown]. Among these groups, 54 % of non-virgins and 24 % of those who transitioned had engaged in sexual intercourse by age 15 [data not shown].

A higher proportion of male adolescents who transitioned reported high levels of father-son communication (48 %) followed by those who are still virgins (43 %). Female adolescents who were virgins reported high father-daughter communication (42 %) followed by those who transitioned (35 %). For both male and female adolescents, the proportion reporting high levels of father-child communication was lowest among non-virgins – 28 % for males and 20 % for females. The associations found for mother-child communication differed compared to father-child communication. More female adolescents than male adolescents reported high levels of mother-child communication. Specifically, approximately 71 % of female virgins reported high levels of mother-daughter communication compared to less than 39 % of male virgins. Additionally, approximately half of female non-virgins and those who transitioned compared to less than a third of male virgins and those who transitioned reported high levels of mother-child communication. A greater proportion of adolescents reported having no father figure (19–36 %) compared to having no mother figure (1–17 %) in their lives.

### Multivariate analyses

Table 3 shows the results of the logistic regression models conducted to identify the correlates of transition to first sexual intercourse among the subset of adolescents reporting no sexual intercourse at Wave 1 (763 males and 744 females). In the first set of models (Models 1 and 3), only parental variables measured in Wave 1 were included as explanatory variables. The second set of models (Models 2 and 4) included both the parental factors and the control variables measured at Wave 1. The models with the control variables had better fit indices for the data compared to models without the control variables.

**Table 3:** Logistic regression models predicting transition to first sexual intercourse, by sex

Model variables	Males (*n* = 763)		Females (*n* = 744)	
	Model 1 OR (95 % C.I.)	Model 2 OR (95 % C.I.)	Model 3 OR (95 % C.I.)	Model 4 OR (95 % C.I.)
Parenting variables				
Parental monitoring				
Low/does not live with parent(s)	1.00	1.00	1.00	1.00
Medium	0.68 (0.33–1.40)	0.82 (0.37–1.80)	0.62 (0.31–1.24)	0.77 (0.34–1.73)
High	0.70 (0.36–1.38)	0.90 (0.41–1.96)	0.37 (0.17–0.78)*	0.56 (0.22–1.47)
Parental discipline				
Scolded (ref = never scolded)	0.80 (0.37–1.74)	1.01 (0.42–2.42)	0.72 (0.32–1.59)	0.74 (0.29–1.85)
Spanked/slapped (ref = never spanked/slapped)	0.29 (0.15–0.56)*	0.74 (0.36–1.52)	0.31 (0.15–0.63)*	0.55 (0.23–1.29)
Communication with father figure				
No father figure	1.00	1.00	1.00	1.00
Low	1.32 (0.54–3.21)	1.15 (0.43–3.05)	0.44 (0.21–0.93)*	0.30 (0.13–0.68)*
High	1.88 (0.83–4.26)	1.89 (0.80–4.45)	0.69 (0.34–1.42)	0.72 (0.34–1.51)
Communication with mother figure				
No mother figure	1.00	1.00	1.00	1.00
Low	0.25 (0.09–0.69)*	0.23 (0.08–0.64)*	0.77 (0.08–7.01)	1.34 (0.23–7.68)
High	0.28 (0.10–0.84)*	0.33 (0.11–0.96)*	0.48 (0.05–4.38)	0.77 (0.14–4.23)
Controls				
Age	---	1.36 (1.13–1.64)*	---	1.29 (1.10–1.51)*
Viwandani (ref = Korogocho)	---	1.25 (0.61–2.54)	---	0.96 (0.46–2.01)
Out-of-school (ref = in-school)	---	3.45 (1.63–7.28)*	---	3.74 (1.84–7.60)*
Peer models for risk behavior^a^	---	1.09 (0.80–1.47)	---	1.32 (0.94–1.86)
Delinquency^a^	---	0.85 (0.51–1.41)	---	1.97 (1.10–3.53)*
Religiosity^a^	---	0.73 (0.55–0.96)*	---	0.92 (0.62–1.35)
Model fit	Wald χ2 (8) = 26.99	Wald χ2 (14) = 53.23	Wald χ2 (8) = 28.50	Wald χ2 (14) = 68.55

Model 1: unadjusted model for males; Model 2: adjusted model for males; Model 3: unadjusted model for females; Model 4: adjusted model for females

^*^Statistically significant at *p* < 0.05

^a^Increasing values indicate higher levels of negative peer influence, delinquency, or religiosity

In Models 1 and 3, high levels of parental monitoring were significantly associated with lower odds of reporting the first sexual intercourse between the two waves for female adolescents but not for male adolescents. Females who reported high levels of parental monitoring were 63 % less likely to transition to first sexual intercourse compared with females who reported low levels of parental monitoring or were not living with a parent or guardian. The association remained non-significant for males and became non-significant for females when age, residence, school enrollment, peer models of risk behavior, delinquency, and religiosity were controlled for.

Male and female adolescents who were spanked or slapped as a form of discipline were less likely to report sexual debut between the two study waves [males OR = 0.29, *p* < 0.05; females OR = 0.31, *p* < 0.05] compared with those who were never spanked or slapped. However, these associations were no longer significant when control variables were added as shown in Models 2 and 4. Being scolded as a form of discipline did not predict transition to first sexual intercourse.

Communication with the father was not significantly associated with transition to first sexual intercourse among the male adolescents but was significant for the female adolescents. Female adolescents who reported low levels of communication with their father figure were 56 % less likely to have transitioned compared with females who reported no communication with their father figure (*p* < 0.05). This association remained significant and in the same direction with the addition of control variables in Model 4 [OR = 0.30, *p* < 0.05]. However, high levels of communication with the father did not predict transition to first sexual intercourse for the female adolescents in our sample (*p* > 0.05). Communication with the mother was found to be significant for only the male adolescents in both models. Specifically, male adolescents who reported low and high levels of communication with their mothers were approximately 75 % and 72 % less likely to commence sexual activity within the study period compared to those who reported no communication with their mothers (*p* < 0.05). These associations remained significant when control variables were taken into account in Model 2.

The adolescents’ age in Models 2 and 4 was significantly and positively associated with transition to first sexual intercourse for both males and females: specifically, a one year increase in age increased the odds of transitioning to first sexual intercourse by 36 % and 29 % for males and females respectively (*p* < 0.05). Being out of school was positively associated with transition to sexual intercourse. Out-of-school male and female adolescents were 3.5 times and 3.7 times more likely to report sexual debut between the waves compared with adolescents who were in-school at the time of the survey (*p* < 0.05). Delinquent behavior was associated with higher odds of initiating sexual intercourse for females but not males. A point increase on the delinquency scale was associated with about twice the odds of transition to first sexual intercourse among female adolescents [OR = 1.97, *p* < 0.05]. This association was not observed for the male adolescents. Conversely, lower levels of religiosity were associated with higher odds of sexual debut between study waves for male adolescents but not for female adolescents. A point increase on the religiosity scale was associated with 27 % lower odds of transition to first sexual intercourse for males [OR = 0.73, *p* < 0.05]. This association was not observed for the female adolescents. Area of residence was not significantly associated with transition to first sexual intercourse for both male and female adolescents in our sample.

## Discussion

Little is known about the association between parenting practices and adolescent sexual behavior in African settings especially in informal settlements or slums, which are becoming more prevalent in urban areas. Previous studies have shown that young people living in resource-poor urban settings are more likely to engage in sexual risk behaviors. However, as noted earlier, with the exception of a handful of studies, we know little about how various aspects of parenting practices influence the transition to first sexual intercourse. Our findings show that parent-child communication was the most significant factor associated with sexual debut, albeit in gendered ways, even after controlling for demographic and psychosocial characteristics. Mother-son communication was significant for adolescent males, while father-daughter communication was significant for adolescent females. We also found that parental monitoring and parental discipline did not predict sexual debut for both male and female adolescents when relevant covariates were controlled for.

Only about 1 in 4 males and 1 in 3 females reported high levels of parental monitoring. Low levels of parental monitoring may reflect lower levels of parental supervision as many adults in urban informal settlements may be employed or looking for work and as such are out of the homes most of the day. Further, we found no association between parental monitoring and transition to first sexual intercourse. This finding is consistent with results from studies conducted in other African countries (Burkina Faso, Ghana, Malawi, and Uganda) [9]. In contrast, studies conducted in the United States (even among urban-poor African American adolescents), Slovakia, and Scotland indicate that high levels of parental monitoring delay sexual debut [12, 22, 37–39]. Therefore, the effect of parental monitoring on adolescent sexual risk behaviors may depend on the sociocultural norms of the geographical context. Programs that aim to involve parents in reducing adolescent sexual risk behaviors should tailor the parental involvement to the sociocultural context of their target population.

Studies have found that positive parent-child communication fosters identification with parental values and may reduce the probability of engaging in sexual activity [20, 22]. In this regard, the majority of the study respondents who remained abstinent in both waves reported higher levels of parent-child communication compared to those who reported their first sexual intercourse between waves. Male adolescents who reported any communication with their mothers delayed their sexual debut; however, no such association was found among the female adolescents. It is possible that because daughters tend to already have closer bonds with their mothers [26, 40], there is little variability among females to detect any significant association. On the other hand, father-daughter communication was found to be associated with decreased odds of female adolescents initiating sexual intercourse; however, communication with the father had no significant association with sexual debut among the male adolescents. Interestingly, the significant association between father-daughter communication and sexual debut appeared to be curvilinear as the effect was only significant for low levels of communication. While the parent-child communication items did not specifically address discussions around sexual behavior, the significant association between sexual activity and communication with the parent of opposite sex, particularly for adolescent girls is noteworthy. Previous studies suggest lower levels of communication around sexual matters with fathers compared with mothers or with parents of the opposite gender [9]. However, the high risk of poor sexual and reproductive health outcomes among adolescents in urban slums may mean that both parents find it necessary to have discussions with their male and female children [15–17]. As such, interventions to improve parent-child communications may need to ensure that both fathers and mothers are targeted.

Adolescents who reported corporal punishment (spanking or slapping) whenever they behaved badly were less likely to report transition to first sexual intercourse within the study period. In contrast verbal discipline (scolding) did not. Other studies have also found that adolescents who anticipate parental discipline are less likely to engage in risky sexual behaviors [14, 20, 33]. Previous studies suggest that the change in behavior as a result of corporal punishment may depend on the cultural context [41]; in settings, such as Kenya, where corporal punishment may be culturally-acceptable and considered an appropriate parenting practice, it is expected that physical punishment can result in a decline of risk behaviors among adolescents. However, it is also important to consider that while the fear of punishment may deter adolescents from engaging in sexual risk behaviors, fear of punishment may also prevent them from having open conversations with their parents about sexual and other sensitive issues. Thus, interventions that enhance parents’ capacity to share their values and guide their children’s behavior while maintaining open communication lines may be warranted.

As observed in previous studies in Zambia and the United States, [25, 42] older male and female adolescents were more likely to initiate sexual intercourse during the one year of follow-up than their younger peers. However, males in mid- and late- adolescence had lesser odds of transitioning to first sexual intercourse compared to those in early adolescence. Previous studies have shown that adolescents’ behavior is significantly correlated with that of their peers [13, 43]. Thus, it is possible that male adolescents who resist peer pressure to engage in sexual intercourse in early adolescence may continue to resist pressure to become sexually active as they grow older. The results of this study also indicate that school enrollment is a protective factor for adolescent sexual risk behaviors for both males and females, though it did not modify the relationship between parental factors and transition to first sexual intercourse. Time spent in school-related activities may reduce opportunities for engaging in risk behaviors. Schooling may also increase the opportunity costs of negative outcomes associated with early sexual intercourse including unintended pregnancies.

### Limitations

This study should be interpreted in light of several limitations. First, about a third of the baseline sample was lost to follow up. This high attrition rate may result in biased estimates especially since the adolescents lost to follow up differed from those who remained in the study on several sociodemographic factors such as age, school enrollment, and living with parents. To minimize the effect of this potential bias, all analyses were weighted based on probability of non-response at Wave 2 [44]. Second, although all efforts were made to reassure respondents about the confidentiality of responses, the use of self-reported sexual behavior data also provides a potential for social desirability bias [45, 46]. Third, we relied on data based on adolescents’ perception of parental practices and communication and lacked the parents’ perspectives of their parenting practices. However, as noted earlier, previous studies have shown a high correlation between youth’s perceptions and parents’ report of parental monitoring [19]. Finally, there was inadequate information on the adolescents’ living arrangements such as privacy in the home or whether they live in single-parent households. Yet, living arrangements may influence parental factors and consequently adolescents’ sexual behaviors.

## Conclusion

Studies on adolescent risk behaviors have been on the rise in the last two decades as incidence of morbidities in this age group due to engagement in risky behaviors increases. Recognizing and examining the factors associated with adolescents’ risk behaviors is important for the development and implementation of effective prevention strategies and/or interventions. Active involvement of parents in these prevention interventions can buttress their effectiveness by creating an enabling environment for the adolescents to implement skills and tools they will gain from the interventions. The findings of our study should inform interventions geared towards delaying sexual debut among unmarried adolescents especially those in urban poor settings. For example, parents can be involved in intervention programs by training them on evidence-based risk-reduction strategies such as effective communication skills especially on sexual/reproductive health issues. There is a need for additional research to further understand how best to involve parents in these adolescent risk-reduction strategies. Further research examining the effects of single-parenthood on adolescents’ sexual attitudes and behaviors would also be insightful. In addition, findings suggest that schooling has protective effects on the youth’s development beyond academic competence and efforts are warranted to ensure that all school-aged youth are enrolled in school.

## Abbreviations

DSA: Demographic surveillance area
HIV: Human immunodeficiency virus
NUHDSS: Nairobi Urban Health and Demographic Surveillance System
STI: Sexually transmitted infections
TTA: Transition to adulthood
WHO: World Health Organization
